# Case of Dry Gangrene of the Foot from Anemia, Terminating Fatally the Tenth Day after Amputation

**Published:** 1862-07

**Authors:** A. P. Phillips

**Affiliations:** Casadaga, N. Y.


BUFFALO

### Medical and Surgical Journal

AND REPORTER.

VOL. I. JULY, 1862. NO. 12

### ORIGINAL COMMUNICATIONS.

ART. I.—*Case of Dry Gangrene of the foot from Anemia, terminating fatally the tenth day after amputation.—By Dr.* A. P. PHILLIPS, *Cassadaga, N. Y.*

The subject of this case, A. B. Fisher, Esq., aged 54 years, presented no marked dyscrasia previous to the inception of the following attack:

The affected limb was the subject of a fracture of both bones of the leg about twelve years ago. The lesion very readily repaired, though before he had acquired the use of the limb he was subject to very acute suffering, attended with tetanus. This, as I learned from the patient, was very effectually controlled by a moderate use of strychnia.

My attention was called to the case about the first of December last. He complained of pain about the theca of the great toe, and his paroxysms of suffering were mostly during the night, and with somewhat marked periodicity, the latter part of the night. Regarding the case as purely neuralgic, I prescribed sulph. morphiæ iii grs. and sulph-quiniæ iii to v grs., three times per diem. This treatment, with the addition of murias ferri, was pursued without any apparent relief.

The patient passed the months of January, February and March, without any very marked increase in severity, when about the first of April I was summoned in great haste as the messenger expressed, "they thought him dying." On approaching his residence, the expression he gave by bounding from his chair to the floor, with foot in hand, seemed too intolerable to endure. He complained of his foot freezing. I immediately ordered warm flannels applied to the parts, and gave him three to five grains of morphine, and repeated this three times, once in two hours, without any apparent effect; also gave repeatedly warm brandy sling but all to no satisfactory relief. When one of the attendants, a sister, came in, she clasped the foot, and to our great joy, after imparting animal warmth from her own hands, gave the most passive relief to the poor sufferer. He slept for four hours when he awoke greatly refreshed from his repose.

The pain during his severe suffering had gradually advanced over the dorsalis pedis artery, rendering its pulsation entirely indistinct, with a leaden discoloration remaining over the entire ball of the foot and the three largest toes. The point of first appearance on the great toe was shrunken, vesicant and dead nearly to the first joint.

I had expressed to the family a desire to consult with Dr. Washburn, of Fredonia, and met him the following day, when the patient was comparatively comfortable, though requiring the continued assistance of animal warmth and vitality imparted from the attendants.

Dr. Washburn's notice of the case confirmed my diagnosis and added to the treatment of iron, (which had been continued from previous time mentioned,) the external use of iodine to the entire leg below the knee.—This was continued for three or four days with a gradual extinction of pulsation, even to the popliteal space, thus precluding all reasonable possibility of recovery without a speedy amputation. I consulted Profs. White and Eastman, of Buffalo, who gave an unfavorable prognosis, but were inclined to regard the reasonableness of an operation from its connection with the local injury sustained years before as its predisposing cause. Accordingly, on the ninth of April, the limb was removed at the upper third of the femur, by Prof. Eastman, Drs. Washburn, Aumock and Thompson, also being present.

The appearance of the femoral artery at its point of division was highly cartilaginous, with a warty excrescence studding its external areolar coat.—Its lower third was much reduced in size, being no larger than a common knitting needle, and the middle third of the popliteal artery was perfectly closed, as were also the anterior and posterior tibial arteries.

The patient bore the operation very pasively under the use of ether, which he afterwards described as more pleasurable than painful.

The day and evening of the operation he had so favorably rallied as to desire his usual nutritious nourishment, beef essence and mutton jelly; very good rest was secured during the night by a mild anodyne; secondary hemorrhage which was feared from the vascular turgescence that was somewhat marked, only tinged the external dressings of the stump.

The third day after the amputation, great depression, inability to move, and loss of sensibility, were complained of throughout the entire half of the body of the affected side, with an intolerance to stimulants and the blandest mucilages. The stump discharged good puss and gave every indication of healing kindly. At this stage I added to the use of per-sulph. ferri, which was advised by Dr. Eastman, strychnia, and administered the beef essence, with laudanum by enema. The patient complained of no suffering whatever, when on the seventh day, coma occurred with stertorous breathing and entire loss of consciousness. From the eighth day to the close of his life, there was the most extreme distortion of the facial muscles, and a firm and unyielding contraction of all the muscles of the right side also. The aspect presented about the neighboring parts of the stump was to me really anomalous; one half of the lower portion of the abdomen, even to the scrotum, on a line extending up the linea alba, nearly as high as the umbilicus, was of one uniform erythematous appearance.—The one-half of the scrotum affected was considerably distended, showing most conclusively the existence of effusion from the degree of anemia present in the case.

